# Recovery of the Pupillary Response After Light Adaptation Is Slowed in Patients with Age-Related Macular Degeneration

**DOI:** 10.3390/jemr18060066

**Published:** 2025-11-10

**Authors:** Javier Barranco Garcia, Thomas Ferrazzini, Ana Coito, Dominik Brügger, Mathias Abegg

**Affiliations:** 1 Department of Radiation Oncology, University Hospital Zurich, 8091 Zurich, Switzerland; 2 Berner Augenklinik, Zieglerstrasse 29, 3007 Bern, Switzerland; 3 machineMD AG, Weyermannstrasse 36, 3008 Bern, Switzerland; 4 Department of Ophthalmology, University Hospital Zurich, Frauenklinikstrasse 24, 8091 Zurich, Switzerland

**Keywords:** age-related macular degeneration, eye tracking, pupil, pupillary light reflex, retinal disease, VR-based eye tracker

## Abstract

**Purpose:** This study evaluates a novel, non-invasive method using a virtual reality (VR) headset with integrated eye trackers to assess retinal function by measuring the recovery of the pupillary response after light adaptation in patients with age-related macular degeneration (AMD). **Methods:** In this pilot study, fourteen patients with clinically confirmed AMD and 14 age-matched healthy controls were exposed to alternating bright and dark stimuli using a VR headset. The dark stimulus duration increased incrementally by 100 milliseconds per trial, repeated over 50 cycles. The pupillary response to the re-onset of brightness was recorded. Data were analyzed using a linear mixed-effects model to compare recovery patterns between groups and a convolutional neural network to evaluate diagnostic accuracy. **Results:** The pupillary response amplitude increased with longer dark stimuli, i.e., the longer the eye was exposed to darkness the bigger was the subsequent pupillary amplitude. This pupillary recovery was significantly slowed by age and by the presence of macular degeneration. Test diagnostic accuracy for AMD was approximately 92%, with a sensitivity of 90% and a specificity of 70%. **Conclusions:** This proof-of-concept study demonstrates that consumer-grade VR headsets with integrated eye tracking can detect retinal dysfunction associated with AMD. The method offers a fast, accessible, and potentially scalable approach for retinal disease screening and monitoring. Further optimization and validation in larger cohorts are needed to confirm its clinical utility.

## 1. Introduction

The pupillary light reflex is triggered by a light stimulus in the retina, and it is then transmitted via intrinsically photosensitive ganglion cells along the optic nerve towards the dorsal midbrain. From there, the signal travels along the parasympathetic pathway back to the pupil. Clinically, this reflex pathway is being used to assess structural integrity along the reflex circuit [1,2,3]. In an unconscious patient, for example, the pupillary light reflex can be used to assess integrity of the brainstem [4]. In ophthalmology, the most common use of the pupillary light reflex is to test for the relative afferent pupillary defect (RAPD), which indicates optic nerve disease [5]. But alterations in the pupillary response have also been reported in numerous neurodegenerative diseases, such as Alzheimer’s disease [6]. With the recent entry of advanced virtual reality headsets equipped with eye-tracking technology in consumer markets, pupil tracking has become available to a broad public. We aimed to explore whether such accessible technology could be used to clinically examine the front end of the pupillary reflex pathway, i.e., the retina, in order to investigate whether the integrity of the retina could be assessed at the level of the pupils [7].

Age-related macular degeneration (AMD) is one of the most common retinal diseases and it is the leading cause of registered blindness in Europe [8]. AMD currently impacts an estimated 20 million individuals in the United States and 196 million globally [8]. By 2040, the number of people affected by AMD is projected to rise to approximately 288 million worldwide [9].

One clinical hallmark in patients with AMD is that they exhibit difficulties with dark adaptation [10,11]. After AMD patients are exposed to a prolonged light stimulus and then exposed to darkness, their retina takes longer in the dark to regain full visual function as compared to healthy subjects. This is probably due to impaired retinal pigment epithelium function which is involved in regeneration of the photopigment in the physiologic visual cycle [12,13].

In this study, we tested the recovery of the pupillary response in darkness after prolonged light stimulation in healthy subjects and in patients with AMD. This is based on the hypothesis that a retinal deficit that causes a delayed recovery of visual function after light exposure also affects the recovery of pupillary function.

## 2. Methods

### 2.1. Participants

In 2021, we included patients of the University Eye Clinic at Inselspital Bern with clinically confirmed AMD with a loss of at least one line of visual acuity, hence a decimal visual acuity ≤0.8. The diagnosis was made during routine clinical workup and was mostly based on optical coherence tomography. We included patients with any disease severity and with dry or wet AMD. A total of 14 people with AMD were included in this study, 5 were considered as early AMD and 9 were considered advanced stage AMD. The distinction of early and advanced stage was arbitrarily made based on a decimal visual acuity of 0.5, meaning that patients with visual acuity of 0.5 or better were considered early and the others as advanced. We also included an age-matched control group composed of 14 individuals, most of them with an anterior segment problem in the untested eye, with no history of retinal disease and a decimal visual acuity of 1.0 measured with number-optotypes at 5 m distance. Only one eye was tested in each participant. 5 out of 14 patients were pseudophakic in the AMD cohort compared to 3 out of 14 in the control group. The study was approved by the local ethics committee of Zürich. Protocol code 2019-02174 and approved on 31 March 2020. All patients and control subjects gave their oral and written consent.

### 2.2. Experimental Setup

We used a virtual reality headset (varjo XR-3 mixed reality headset, Helsinki, Finland) to control light exposition to the eyes and to record pupil size. The pupils were recorded with a video-based pupil-tracker running at 200 Hz, which is integrated in the headset.

**Figure 1:**
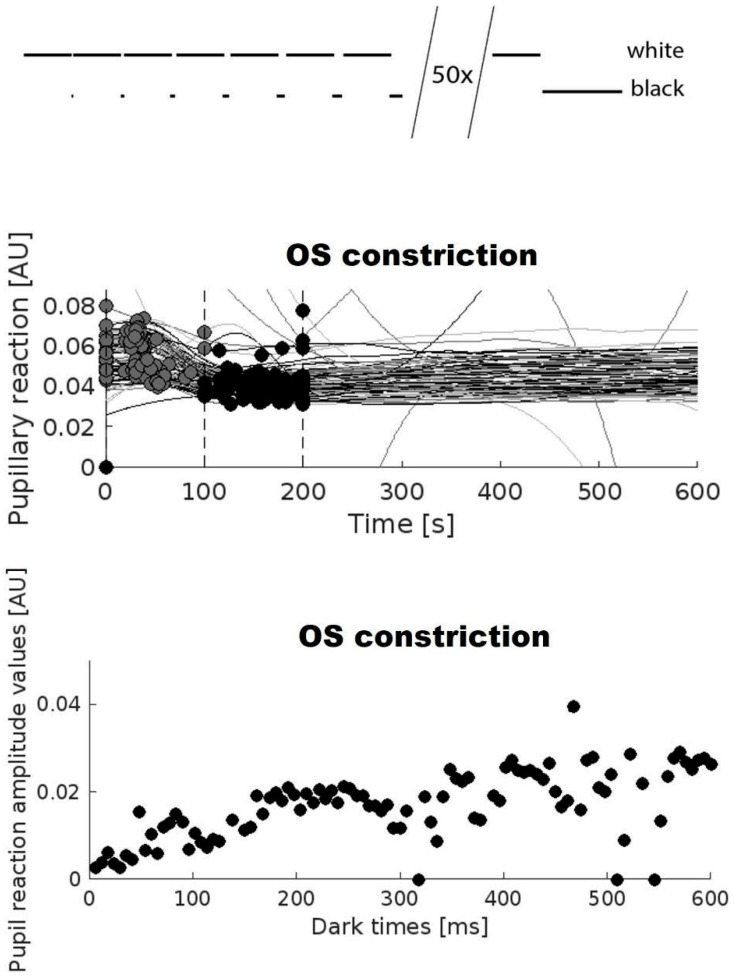
Upper panel shows the stimulation protocol used for assessing the recovery of the pupillary light response after light exposure. 50 repetitions of bright (white) and dark stimuli (black) are applied in one eye with an increase in the duration of darkness in each cycle. Middle panel shows the overlay of 50 measures of the PLR after re-onset of light in one patient. The region of interest that was used to find the smallest pupil size and the region of interest used to identify the largest amplitude are indicated. The difference between the two corresponds to the PLR amplitude. Circles indicate pupillary size before and after re-onset of light, the difference corresponds to the numerical amplitude of the pupillary light response. Lowest panel shows a scatter plot with magnitude of the PLR on the y-axis and duration of the dark stimulus on the x-axis for a single healthy subject. PLR: Pupillary Light Reflex. [AU]: arbitrary units.

The pupil adaptation protocol consisted of setting the display luminosity to minimum in the untested eye. The luminosity of the display used to measure the tested eye alternated between maximum screen brightness (white, luminance = 150 Nits) and darkness (black, luminance < 1 Nit). The white screen corresponds to a luminance of 150 Nits. A total of 50 cycles of bright/dark alterations were made (Figure 1). The white always lasted 3 s, followed by black, which increased the duration by 100 ms in each cycle. Thus, 3s of white was interrupted by 100 ms of black in the first trial. This was followed by 3s of white, followed by 200 ms of black and so on. After 50 cycles with an increase of 100 ms per cycle, three single cycles were performed: 3 s white, 7 s black; 3 s white, 10 s black and 3 s white, 15 s black. At the end of the experiment, the measuring procedure started again with a white screen of 3s followed by a black screen during 100 ms, this was repeated 10 times in each participant.

### 2.3. Analysis

We determined the minimal pupil size in a region of interest (ROI) between 100 and 200 ms after re-onset of light for each cycle and the maximal pupil size in the time between 0 and 100 ms after re-onset of white screen (see vertical dashed line in middle panel of Figure 1). The difference between these two values was defined as amplitude of the pupillary response (Figure 1).

To further investigate the differences in pupillary constriction between AMD patients and healthy controls, we employed a linear mixed-effects model using MATLAB’s fitlme function (MATLAB release 2023b, MATHWORKS, 1 Apple Hill Drive, Natick, MA 01760-2098, USA). Our analysis was based on 1666 observations, with data collected over a 500 ms period with 100 ms intervals, resulting in 50 data points per eye for each participant. We preprocessed the data by calculating the average constriction amplitude between left and right eyes for each participant at each time point.

Our model included fixed effects for time and group (AMD vs. Control), as well as random effects to account for individual variations in the form of:

Y = Xβ + Zb + ε,

where Xβ represent the fixed effects, *Zb* the random ones and finally, ε is the error.

To evaluate the dependence of the pupillary light reflex (PLR) on the group (AMD or Control), the relation is as follows Amplitude ~ 1 + Time + Group + (1|Group). This equation evaluates how the pupillary constriction amplitude is influenced by fixed effects for time and group, while also incorporating a random intercept for group to account for any variability specific to each group. This approach allows for a comprehensive understanding of how both temporal factors and group differences affect the PLR.

To extend our analysis, we also incorporated Age as a variable and examined its interaction with Group. Being the relation now, Amplitude ~ 1 + Age + Time + Group + Age × Group + (1|Group). This extended model investigates the influences on pupillary constriction amplitude from age, time, and group (AMD vs. control), while also considering differences between groups. The interaction term Age × roup allows for the examination of how the effect of age on constriction amplitude varies between AMD patients and controls. The random intercept for group accounts for any unexplained variability at the group level, enhancing the model’s robustness. Additionally, we performed an ANOVA using the marginal tests method with residual degrees of freedom to assess the significance of our predictors in both cases.

To complement these analyses, we explored the use of a Convolutional Neural Network (CNN) to classify individuals as either AMD or control based on their constriction amplitude data. The CNN architecture was designed to process the temporal dynamics of the constriction amplitudes, with layers specifically optimized for feature extraction and classification. The model included convolutional layers with ReLU (Rectified Linear Unit) activations to extract hierarchical features. ReLU introduces non-linearity by outputting the input directly if positive, and zero otherwise, helping mitigate the vanishing gradient problem. Max-pooling layers were used for dimensionality reduction, and dense layers to capture complex relationships in the data. Dropout layers were incorporated to mitigate overfitting during training.

The classifier was evaluated using a five-fold cross-validation strategy to ensure robustness and generalizability. Two setups were tested: the first utilized constriction amplitudes as the sole input features, while the second incorporated both constriction amplitudes and age. Input data was preprocessed through standardization, and a one-hot encoding scheme was applied to the binary labels for compatibility with the network’s output layer. The implementation was carried out in Python (version 3.12), utilizing TensorFlow, Training was performed using the Adam optimizer with categorical cross-entropy as the loss function, and early stopping was employed to prevent overfitting by monitoring validation loss during training.

## 3. Results

**Figure 2:**
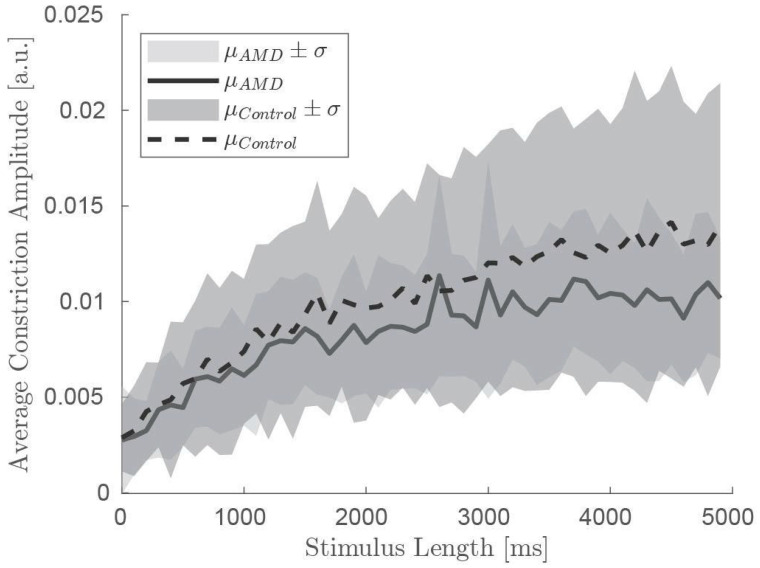
Summary plots showing the recovery of the pupillary light reflex, i.e., an increase in pupillary response with increasing duration of darkness. Healthy controls (grey line) recover faster than patients with age-related macular degeneration (black line). Standard deviation for each group is indicated in shades of grey. [a.u.]: arbitrary units.

Brightness as defined by a white screen at maximum intensity in the VR-goggles that was briefly interrupted by darkness, defined by black screen, readily elicited a pupillary response after re-onset of brightness. This PLR-amplitude increased with increasing duration of the darkness prior to re-onset of brightness (Figure 2). This increase is described here as “recovery of the PLR” with increasing duration of darkness.

Next, we wanted to find out whether PLR recovery was different in healthy and AMD patients. For this, we used a linear mixed model fit by machine learning with dependent variables PLR amplitude. We first analyzed the data without considering age, including fixed effects for time and group (AMD vs. control), as well as random effects for individual variation. The model demonstrated a good fit, with AIC and BIC values of −10,926 and −10,899, respectively. Significant differences in pupillary constriction were found between AMD patients and controls (β = −0.001676, *p* < 0.001), with AMD patients showing lower constriction amplitudes. Additionally, the model revealed a significant effect of time (β = 1.6734 × 10^−6^, *p* < 0.001), indicating an increase in constriction amplitude over time in both groups. The intercept for baseline constriction amplitude was estimated at 0.0059487 (*p* < 0.001), and the model effectively accounted for individual variability, as indicated by the low residual standard deviation of 0.0047761.

We further confirmed the significance of these predictors using ANOVA, which provided the following results: Intercept: F (1, 1390) = 443.96, *p* < 0.001; Time: F (1, 1390) = 356.24, *p* < 0.001; Group: F (1, 1390) = 42.882, *p* < 0.001.

We extended our analysis by incorporating age as an additional variable to explore its influence on pupillary response. In this model, we included fixed effects for time, group, age, and an interaction between group and age. The inclusion of age further refined the analysis, with the group effect remaining significant (β = −0.0086203, *p* < 0.001), confirming that AMD patients still exhibited lower constriction amplitudes compared to controls. The time effect persisted as well (β = 1.247 × 10^−6^, *p* < 0.001), indicating an increase in constriction amplitude over time.

Age had a significant negative effect on pupillary constriction (β = −0.00011093, *p* < 0.001), suggesting that constriction amplitude decreases with age in both groups. Interestingly, the interaction between group and age was also significant (β = 0.00010421, *p* < 0.001), indicating that the age-related decline in constriction amplitude was less pronounced in AMD patients compared to controls.

ANOVA results for this model confirmed the significance of all terms: Intercept: F (1, 1388) = 374.76, *p* < 0.001; Time: F (1, 1388) = 163.73, *p* < 0.001; Group: F (1, 1388) = 111.6, *p* < 0.001; Age: F (1, 1388) = 153.05, *p* < 0.001; Group–Age Interaction: F (1, 1388) = 79.364, *p* < 0.001. These results show the interplay between age and disease status in shaping pupillary response, suggesting that while age impacts both groups, its effect is moderated by the presence of AMD.

To further complement our statistical findings, we employed a CNN-based binary classifier to differentiate between AMD and control groups using constriction amplitude data as input. The model was evaluated using a five-fold cross-validation approach to ensure robustness. When trained using only constriction amplitudes, the CNN achieved a mean accuracy of 92.67% (±9.04%), mean sensitivity 0.90 and mean specificity 0.733, demonstrating reliable performance across the validation folds. The confusion matrices consistently indicated high sensitivity and specificity for both AMD and control classifications, highlighting the model’s robustness in identifying group-specific patterns in PLR data.

## 4. Discussion

This study demonstrates that the recovery of the PLR after exposure to brief dark stimuli, i.e., dark adaptation, differs significantly between healthy individuals and patients with age-related macular degeneration (AMD). Specifically, the amplitude of the PLR increases with longer dark exposures, but the recovery rate is significantly slower in AMD patients. This finding suggests that at least a part of the recovery rate is the result of regeneration of the photopigment in the photoreceptor layer of the retina. This novel observation highlights the potential of pupillometry as a tool for detecting retinal dysfunction, leveraging commercially available virtual reality (VR) headsets equipped with eye-tracking technology.

In addition, using constriction amplitude as input for a CNN-based binary classifier, we could differentiate between AMD and control groups with high accuracy (92%), sensitivity (90%), and specificity (70%). This underscores the potential of PLR metrics, more specifically the difference in dark adaptation, in AMD detection. It also underlines the potential of CNNs in PLR analysis, showing that the temporal features of constriction amplitude data contain strong, distinguishable patterns. The model’s performance, even in the absence of age information, further emphasizes the discriminatory capacity of PLR amplitude dynamics for identifying AMD. This machine-learning-based approach offers a complementary perspective to the statistical models and highlights the synergy between traditional and modern analytical techniques for understanding the pupillary response in AMD.

The observed slower PLR recovery in AMD patients aligns with established pathophysiological mechanisms. AMD is characterized by the dysfunction of retinal pigment epithelium (RPE) and photoreceptor degeneration, both of which are crucial for the regeneration of photopigments such as rhodopsin and melanopsin (12). Previous studies have shown that delayed dark adaptation is a hallmark of AMD, particularly in its early stages, where impairment in RPE-mediated chromophore recycling slows the visual cycle, and with studies showing that rod-mediated dark adaptation is among the best visual functions to discriminate among healthy eyes, early AMD, and intermediate AMD eyes [14,15,16]. Indeed, the relationship between AMD and delayed dark adaptation has been supported by research showing that abnormal dark adaptation in older adults with normal macular health is associated with an increased risk of developing early AMD within three years, which suggests that impaired dark adaptation may be an early functional biomarker for AMD risk [16].

Previous studies have shown that rod and melanopsin-driven intrinsically photosensitive retinal ganglion cells (ipRGC) contributions to the PLR are altered in early AMD [17,18], and that the post-illumination pupil response is impaired in these patients [17]. Other studies investigating PLR in AMD showed alterations in several pupil parameters such as latency, amplitude, time for maximum constriction, maximum constriction velocity, maximum constriction acceleration, time for maximum velocity, and pupil radius when compared with a control group [19,20,21,22,23]. A previous study suggested that pupil assessment could be valuable for objectively assessing retinal function in AMD [21]. The delayed recovery of PLR in our current study provides further evidence that pupillary metrics can reflect retinal biochemical processes, offering a non-invasive and non-expensive proxy for assessing retinal health. Since the PLR is triggered by both an activation by photoreceptors conveying the signal to ipRGCs and the photopigment within ipRGCs, a lesion in the photoreceptors will affect the PLR. Rather than the amplitude of PLR evoked after long dark exposure, which is dependent on a large variety of factors, we used the rate of recovery of the PLR during darkness. We believe that this metric directly reflects the speed of the biochemical processes that transform all-trans retinal to 11-cis retinal and the analogous process of the photopigment in ipRGCs.

The use of VR technology in this study provides a significant advantage over traditional pupillometry setups. VR headsets with integrated eye-tracking capabilities offer precise control over visual stimuli and real-time monitoring of pupil size. This accessibility opens the door to broader applications, including remote or home-based screening for retinal diseases. Prior work has demonstrated the utility of VR-based eye-tracking devices for neurological and ophthalmological assessments [24]. This study extends this utility to the detection of AMD, suggesting a paradigm shift in how retinal diseases may be diagnosed and monitored outside clinical settings.

Despite its strengths, the study has limitations that warrant discussion. The small sample size (14 AMD patients and 14 controls) restricts the generalizability of the findings. Moreover, most AMD patients in this study had advanced disease, limiting the applicability of the results to early-stage AMD where diagnostic uncertainty is greatest. Additionally, the absence of an independent validation cohort restricts the ability to fully assess the model’s performance in diverse real-world clinical settings. Finally, the reliance on constriction amplitudes alone, while effective, may overlook other potentially informative features, such as dynamic pupillary response characteristics or additional physiological parameters. While the convolutional neural network (CNN) achieved high diagnostic accuracy, the model’s performance must be validated in external datasets to ensure its robustness and clinical utility.

Although the current study focused primarily on patients with clinically confirmed AMD, including those at advanced stages, our findings raise important possibilities for early or even preclinical disease detection. Dark adaptation abnormalities, particularly delays in rod-mediated recovery, have consistently been identified as some of the earliest functional deficits in AMD, even before visible structural changes occur on imaging [16,17]. Since our method captures slowed pupillary recovery after light adaptation—a process influenced by both photoreceptor and retinal pigment epithelium function—it is plausible that subtle delays in pupillary recovery might also be measurable in individuals at risk for AMD or in the earliest stages of the disease.

This perspective may also help explain the observed specificity of 70%. While promising, this value may have been impacted by the inclusion of patients across a wide spectrum of AMD severity. In particular, combining early and advanced stage patients into a single “AMD” category may have introduced variability in the response range, potentially blurring the boundary between the patient and control groups. Additionally, some individuals in the control group may have had undiagnosed subclinical changes or preclinical AMD, especially given that dark adaptation deficits may precede fundus abnormalities by several years [16]. This could have led to misclassification by the machine learning model, lowering specificity.

Addressing these issues will require well-designed future investigations. Future studies should aim to recruit larger and more phenotypically diverse cohorts, including individuals with varying severities of AMD (e.g., early, intermediate, and advanced), those with genetic or lifestyle risk factors, and patients with other retinal diseases such as diabetic retinopathy or retinal vein occlusion. Stratifying participants by disease stage and incorporating multimodal features—such as retinal imaging or genetic profiles—may help improve both specificity and clinical relevance. Longitudinal follow-up will be essential to determine whether pupillary recovery metrics can predict clinical progression, monitor disease over time, or serve as early biomarkers prior to structural retinal changes. External validation and expansion of the feature set could further strengthen the model’s robustness and generalizability.

In the context of clinical practice, the proposed PLR recovery test may complement existing diagnostic modalities such as optical coherence tomography (OCT) and fundus photography. However, its greatest utility might lie in non-specialist settings, where fundus imaging is currently not feasible. For example, general practitioners equipped with VR headsets could use this test to screen for AMD in at-risk populations, such as older adults with a family history of retinal diseases. Similarly, home-based testing could facilitate early detection, allowing timely referrals to ophthalmologists.

In conclusion, this pilot study establishes proof-of-concept that PLR recovery metrics can differentiate AMD patients from healthy individuals. By leveraging accessible VR technology, this approach holds promise for expanding the reach of retinal disease screening and monitoring. However, larger studies and validation efforts are necessary to translate these findings into routine clinical practice. The integration of advanced analytics and multimodal assessments may further enhance the diagnostic and prognostic utility of this novel testing approach.

## Data Availability

The raw data supporting the conclusions of this article will be made available by the authors on request.
